# Health neuroscience 2.0: integration with social, cognitive and affective neuroscience

**DOI:** 10.1093/scan/nsaa123

**Published:** 2020-09-05

**Authors:** Tristen K Inagaki

**Affiliations:** Department of Psychology, San Diego State University, San Diego, USA

Health is a primary concern, whether it is our own health or the health of friends and family. This is never more apparent than when threatened. The current pandemic provides a sad reminder of just how vulnerable our health can be. Researchers share this concern. Even the founders of modern psychology, including William James and Willhelm Wundt, were originally trained in medicine and had interests in health before turning to Psychology. Understanding health is, therefore, interconnected with the goals of Psychology. Yet health, defined nearly 75 years ago by the World Health Organization (1948) as a complete state of physical, mental and social well-being and not merely the absence of disease or infirmity, continues to be an elusive and, sometimes, poorly understood phenomenon.

Health Neuroscience, articulated as its own field in 2014 (Erickson *et al.*, 2014), is defined by its explicit focus on understanding how the brain affects and is affected by physical health. In this way, health neuroscience emphasizes the reciprocal ‘cross-talk’ between the brain and other aspects of the individual—the rest of the body (e.g. Garfinkel *et al.*, 2014; Kraynak *et al.*, 2019), the health behaviors and decisions we engage in (e.g. Erickson *et al.*, 2011; Falk *et al.*, 2011; Berkman, 2018), the people and society with whom we interact (Eisenberger and Cole, 2012), the environment within which we live (e.g. Calderon-Garciduenas *et al.*, 2002). From these foci come three overarching goals: to understand the brain (i) as a predictor of health, (ii) as a mechanism linking social and affective experience with health and (iii) as a health outcome in and of itself.

This Commentary briefly reviews where health neuroscience comes from and why it was formed, what the field looks like today and recommendations for what health neuroscience could look like tomorrow. Special emphasis is placed on how readers of *Social Cognitive and Affective Neuroscience* (SCAN) can contribute to the field.

## Foundational perspectives

Well before the invention of modern brain imaging techniques, there has been a deep appreciation for the brain’s inseparable connection to aspects of physiology, psychology and behavior that contribute to health (e.g. James, 1890). Health neuroscience, however, represents the intersection of more contemporary research traditions: cognitive and affective neuroscience, health psychology, behavioral medicine, epidemiology and public health. From neuroscience, health neuroscience borrows perspectives that integrate peripheral physiological responding into their understanding of the function and structure of the brain. From health psychology, health neuroscience builds on the biopsychosocial approach to health—an approach in which biological responding, psychological processes and the social environment share equal footing and interact in meaningful ways to influence health. Behavioral medicine and epidemiology bring an emphasis on mechanistic mediators, treatment targets and predictors of health and its determinants at a large scale (e.g. globally, across a country or a neighborhood). And from public health, health neuroscience borrows emphases on preventative health behavior and an understanding of individuals as existing within a community, healthcare and health policy systems. Closely allied fields, such as population neuroscience and translational neuroscience, share similar origins (Falk *et al.*, 2013; Woo *et al.*, 2017; Berkman, 2018).

## Why health neuroscience?

Despite Paul Maclean’s early model of the brain—namely, the ‘limbic system’—as a substrate by which affective processes relate to chronic illness (MacLean, 1949), inclusion of the brain was either absent from or implicit in later influential models of human health (e.g. Ajzen, 1985; Rosenstock *et al.*, 1988; Miller *et al.*, 2009; Del Giudice *et al.*, 2011). Regardless of the reasons, its conceptual absence lead to far-reaching consequences for health research in the psychological and behavioral sciences.

If the intent of a theoretical model is to guide research by generating testable hypotheses one is, of course, more likely to test a hypothesis about predictors, processes or outcomes that are in a model than those that are not in the model. Exclusion of the brain, therefore, might have led to the conclusion that the role of the brain in health is ignorable. The biopsychosocial approach to health, as one overarching example, became popular because of its goal to understand how psychological factors ‘get under the skin’ to influence physical health. Though still a popular term, the idea that psychology ‘gets under the skin’ suggests that psychological experience somehow sneaks into the body. In reality, psychological experience walks straight through the front door—the brain. The exclusion of the brain, however, deemphasizes this fact and the brain’s role in health.

Without conceptual inclusion and integration of the brain, sophisticated hypotheses about the brain’s role in health, particularly physical health, have been lacking. Indeed, conceptual inclusion of the brain in health necessitates a nuanced understanding of the brain that is more than an empty placeholder that sends predictive signals and receives inputs from the periphery. A related consequence is that conceptual integration of the brain with the rest of the body and broader context (e.g. one’s culture, environment, psychological experience) is still poor—a fact that stands against the desire to have a holistic understanding of health. In other words, exclusion of the brain leaves a fully articulated pathway by which psychological experience affects the body or how the body affects psychological experience, behavior and decision-making incomplete. Ignoring the brain at the theoretical level, therefore, trickles down to research implementation and moves understanding of the brain’s role in physical health, in particular, behind that of mental health.

A broader consequence of excluding the brain is that the rigorous training needed to measure brain structure and function and link brain measures with other levels of analysis (e.g. branches of the autonomic nervous system) is also rare. Formal training programs outside of individual labs that emphasize both psychology and physical health, for example, exist in only a handful of institutions and remain rare at the departmental and institution levels. This fact might appear surprising given the numerous resources dedicated to health worldwide (private and public funding, research societies, journals and textbooks). Together, the exclusion of the brain in theoretical models of health and dearth of formal training are particularly detrimental for discovery with brain measurement methods—which require a high level of expertise and substantial financial burden to conduct.

Stepping away from the research itself, real change to individual, community and population health requires the attention of policy-makers. Research has shown that information is perceived to be more believable and credible when it is seemingly based on neuroscience and incorporates measures of the brain (McCabe and Castel, 2008; Weisberg *et al.*, 2008; cf Farah and Hook, 2013). The onus is, therefore, on the research community to demonstrate physical connections between the brain and health and then to manage this messaging once it reaches the level of policy. As an example relevant to all readers of SCAN, fMRI and EEG studies can be utilized to enhance the credibility of claims that social and affective experience bidirectionally influence physiology. Such connections are enormously meaningful for health.

Brain research also produces new knowledge that complements other evidence on social and public health problems A clear example is the pervasive impact of socioeconomic disadvantage on the brain across the lifespan (McEwen and Gianaros, 2010; Farah, 2018). Such knowledge adds to what is already known about the patterning of chronic health problems at the forefront of policymaker’s minds (e.g. hypertension, type II diabetes, cancer and now coronavirus disease of 2019 [COVID-19]). Brain research helps us understand how these socially patterned health problems might be taking an added toll on the brain to confer risk for preventable adverse outcomes that are amendable to policy change.

## Health neuroscience 2.0

Since health neuroscience first took formal shape, a number of important steps have been made. We have defined a research space in which the brain and physical health share bidirectional influence and the three goals—to understand the brain as a predictor, mechanism and outcome—have been articulated. The following starts the journey toward Health Neuroscience 2.0 by revisiting the original three goals of health neuroscience.

### The brain as a predictor of health

There is a long tradition within health psychology and medicine to use biomarkers to predict health in order to reveal points of intervention, intermediate outcomes and risk stratifiers. Such a tradition dovetails with an increasingly appreciated, if not yet widely accepted, perspective of the brain as a predictive organ (Berkman and Falk, 2013; Barrett, 2017; Woo *et al.*, 2017; Gianaros and Jennings, 2018). The switch from previous understanding of the brain as solely reactive to predictive represents a paradigm shift in neuroscience and one that lends itself to one of the major goals of health neuroscience: using the brain to predict health and prevent disease. Illustrative examples from the current special issue that capitalize on this perspective include those that use state-of-the-science analytical approaches (e.g. machine learning to identify multivariate patterns of neural activity) to predict a host of the most pressing health issues facing society today: cardiovascular disease (Gianaros *et al.*, 2020), obesity (Stice *et al.*, 2019; Cosme *et al.*, 2020; Donofry *et al.*, 2020; Verstynen *et al.*, 2020) and physical pain (Reddan *et al.*, 2020). Similar approaches help us understand links between brain patterns of activity to emotional content and systemic inflammation, a key biological mediator linking psychological experience and health (Alvarez *et al.*, 2020), or to health messages and population-level sharing of the information (Dore *et al*., in press). And supplementing the use of task-based imaging, there is also promise in examining links between resting state brain connectivity and health-relevant outcomes (Inagaki and Meyer, 2019; Mehta *et al.*, 2019).

Prediction of health outcomes from neural activity, however, has also proven difficult (e.g. Gianaros *et al.*, 2020; Cosme *et al.*, 2020), providing room for a number of future directions. At the most fundamental level, what health-relevant physiological responses are best predicted by brain activity? And, what boundary conditions (e.g. when and for whom) exist for reliable prediction? Moving toward a within-person, lifespan perspective on prediction, does the brain’s response to socio-emotional experience predict response to treatment, willingness to engage in preventative health behavior, or the progression of chronic disease within a person? At the broadest level, will the brain as predictor approach help us extend healthy years and slow the time in which disease negatively impacts function?

### The brain as a mechanism bridging social-affective experience and health

Decades of research from epidemiology and public health have established that social and affective experiences are key physical health determinants. As examples relevant to all readers of SCAN, objective social indicators like social network size and subjective indicators like feelings of loneliness are robust predictors of health and mortality (Cacioppo and Hawkley, 2003; Christakis and Fowler, 2007; Holt-Lunstad *et al.*, 2010). Yet, we know surprisingly little about the neurocognitive processes linking social indicators with health. Social neuroscience, in particular, is well positioned to unpack these relationships in terms of brain mechanisms.

In the current special issue on health neuroscience, the brain is examined as a mechanism linking psychological stress and inflammation in cancer patients (Leschak *et al.*, 2020) and early-life trauma, inflammation and symptoms of PTSD and depression in African American women (Mehta *et al.*, 2019). Others assess basic mechanisms in healthy samples (a prevention oriented approach)—the brain’s response to painful or emotionally salient content as a mechanism underlying self-affirmation (Dutcher *et al.*, 2020), support-giving (Inagaki and Meyer, 2019) and supportive touch’s effect on health (Reddan *et al.*, 2020). Finally, Poulton and Hester, 2019 review brain mechanisms contributing to risky decision making and the development of substance use disorder.

A noticeable missing piece in the goal to understand the brain as a mechanism is that, for the most part, mechanism is implied, but not tested or manipulated. For instance, resting state connectivity between brain regions and task-based activation in the dorsal anterior cingulate, anterior insula and amygdala in response to people in need are negatively related, suggesting that social experience might contribute to health by altering activity in these regions (Inagaki and Meyer, 2019). Mediation, however, was not directly shown. Future research could remedy this issue by directly testing mediation using the statistical techniques and experimental methods common to psychologists (e.g. as shown in Muscatell *et al.*, 2016). For example, one could directly assess whether the neural processes widely studied in social neuroscience (prejudice, social influence, the self, social comparison, social norms, obedience and conformity, self-regulation, aggression, close relationships, etc.) mediate associations between stress (Cohen *et al.*, 2016), social status (Matthews and Gallo, 2011; Cundiff *et al.*, 2020) or social ties and health (Holt-Lunstad *et al.*, 2010).

### The brain as a health outcome

Current understanding of the brain as a health outcome comes largely from the area of population neuroscience. Research in this area has identified differential brain patterns depending on socio-economic status, social network size and neighborhood (Falk *et al.*, 2013; Gianaros *et al.*, 2017). Additional emphases are on the negative effects of hypertension, metabolic syndrome, diabetes and poor sleep on the brain. Social, cognitive and affective factors remain important in all of these areas, but are wide open for additional research. As highlighted in Cardenas *et al.*, 2019, one area ripe for further research is how pregnancy affects the structure and function of the brain itself. Other examples include whether repeated experiences of social rejection and peer victimization early in life alter the brain in ways that confer risk for suicide or self-harm later in life (Olié *et al.*, 2017). These, and the questions posed above, are all critical questions to be addressed in future health neuroscience research.

## New and continuing emphases: where can the SCAN community contribute to health neuroscience?

With the world’s health threatened by the COVID-19 pandemic and the stark reminder of long-standing health disparities in the USA, now is a pressing time to care about physical health. A major strength of health neuroscience is its collaborative, interdisciplinary nature. In an effort to nourish the norm to collaborate, this final section highlights specific areas in which readers of SCAN can contribute to our understanding of physical health and help shape health neuroscience 2.0 moving forward. The commentary concludes with continuing emphases for current contributors to the field.

### Embed the brain in psychological experience

Many studies in health neuroscience, including those in the current special issue, adopt an individual difference and correlational approach to their research questions rather than an experimental approach. The cross-sectional approach stems from: (1) the field’s close ties to epidemiology and (2) constraints around which health concerns and outcomes are studied. Until the recent pandemic, non-communicable (chronic) disease—cardiovascular disease, cancers, respiratory disease, diabetes—has remained the top cause of death globally for more than a decade (World Health Organization, 2016). Similarly, the gold standard biological measures are those shared with the field of medicine and allied fields (kinesiology, physiology, etc.) including outputs of the autonomic nervous (parasympathetic and sympathetic divisions), endocrine and immune systems. Measures like visceral fat, rather than body mass index (BMI) or actigraphy or maximal oxygen uptake (V02 max), rather than self-reported physical activity, are the gold standard measures from fields like physical activity and sleep.

The (accurate) perception that the health issues and health outcomes are rigidly defined produces one of the greatest tensions between those trained in psychology and the social sciences and those trained in medicine—a tension between the desire to advance theory and a desire to advance clinical practice. This tension produces a goal so lofty that simply demonstrating a correlation between two outcomes (e.g. loneliness and inflammation) that fulfills both goals (i.e. is theoretically interesting and measured with the appropriate level of consideration for the factors that contribute to any one outcome) is often a significant contribution. As a consequence, the field remains rife with cross-sectional findings.

For a psychologist, it might even appear as if there is no room for creativity or innovation—the health problems and outcomes are what they are, the standard tasks are what they are—and that an experimental approach offers little value. Contrary to this perception, social and affective neuroscientists can make significant contributions to health neuroscience by bringing experimental approaches to the field using manipulations that better approximate real-world experience. Recent pushes in social neuroscience to assess the brain with naturalistic experience (Schilbach *et al.*, 2013), such as investigating how people connect during social interaction (Parkinson *et al.*, 2018), similarly lends itself to filling large gaps in understanding of how social factors and health bidirectionally influence one another. In paying more careful attention to the face validity of our paradigms, we can remedy the sterile read of a largely correlational literature and reinvigorate the goal to understand mechanisms.

Using face-valid manipulations is important, not for the sake of innovation or creating ‘cute’ manipulations (an accusation often lobbied at social psychologists), but for clarifying why demographics (race and ethnicity, gender, age) are still some of the most robust predictors of health. Demographic predictors cannot be experimentally manipulated, but the proxies for demographic predictors—psychological experience—can and should be manipulated. It is surprising that experimental approaches are still underutilized in health neuroscience when immersive, self-relevant experimental versions of these experiences—overt and implicit racism, job and interpersonal loss, social support, connection and isolation—can and have been experimentally manipulated in elegant ways within the confines of the scanning environment. Indeed, such real-world experiences drive the effects mistakenly attributed to demographic factors in the broader literature (e.g. racism, rather than race, leads to disparities in health, Volpe *et al.*, 2019). Paradigms that transport real-world relationships into the scanner or induce social emotions, thinking and decision-making have existed in the social neuroscience literature for some time, but are underutilized in health neuroscience (for a summary, see Lieberman, 2010, but also note the leaps forward that the field has taken).

As a specific example from the current special issue on health neuroscience, decades of research from human neuroimaging uses standardized sets of emotional images in order to elicit activity in affect-related brain regions (medial prefrontal cortex, anterior cingulate cortex, anterior insula, amygdala, etc.). More than half of the empirical studies in the current special issue use some version of ‘emotional’ static images (Inagaki and Meyer, 2019; Stice *et al.*, 2019; Alvarez *et al.*, 2020; Cosme *et al.*, 2020; Gianaros *et al.*, 2020; Leschak *et al.*, 2020). Standardized images have undoubtedly produced valuable information. However, there is also room to embed the brain back into psychological experience, to embed the brain back into the everyday mind. Anyone who has experienced the joys and struggles of parenthood, friendship or a job knows that viewing static images of strangers merely scratches the surface of their experience. And whether the brain’s response to these richer social experiences, rather than a posed picture of an ‘angry’ face, confers risk for the development of cardiovascular disease, cancer, obesity or addiction remains often hypothesized, but rarely tested open questions. We cannot conclude that brain activity in response to socio-emotional content does or does not relate to or influence physical health until we create actual emotional responses in our participants (Lieberman, 2019). Similarly, we do not know whether physical illness influences responses to real-world experience (i.e. bottom up processes), from the mundane to the extraordinary, until we more closely approximate these experiences in experimental settings. No other scientists within health neuroscience will dive in with the appropriate level of expertise and interest as the readers and contributors of SCAN.

### The body does not care about theory: how to think about health

While embedding the brain back into psychological experience, an important reminder for those entering health neuroscience is that the body functions outside of the boxes and arrows of a theoretical model. And so, when thinking about or measuring health, constrain conceptual models to systems that actually interact and ask yourself whether the hypothesized outcome exists in a meaningful way in real life. Collaboration and discussion with those in medicine can be tremendously helpful with this part of theory development. In particular, health neuroscience has moved away from a single biomarker approach and a ‘more is bad, less is good’ view and toward a systems approach—an approach in which there is cross-talk between and among levels of analysis in biologically plausible ways (for examples, see Leschak *et al*., Mehta *et al*. or Gianaros *et al*. from the current special issue).This view parallels movement away from the focus on single brain regions in neuroscience (Poldrack *et al.*, 2017; Woo *et al.*, 2017).

Take the outdated example of cortisol, an output of the hypothalamic pituitary adrenal (HPA) axis that is still widely known in psychology as a ‘stress-relevant outcome’. A reasonable hypothesis, based on previous thinking, would be that more cortisol in response to a stressful event would be bad, whereas less cortisol would be better for health. Few within health neuroscience today equate cortisol with stress in such simplistic terms but rather have a more rounded understanding of cortisol’s functions and connection to psychological experience, and a healthy skepticism for its ability to predict health (Crosswell and Lockwood, 2020). Instead, and consistent with the emphasis on interactions across systems, there is an updated appreciation for relationships across markers of both the HPA-axis and the immune system (e.g. glucocorticoid resistance hypothesis; Sapolsky *et al.*, 1986). Similar updated views of measures like BMI (Piché *et al.*, 2020) and markers of biological aging (Levine, 2019) are emerging that highlight the need to treat biomarkers of health with a greater level of complexity.

### Think bigger about the brain

A systems approach to any outcome is, admittedly, a work in progress (Poldrack *et al.*, 2017; Woo *et al.*, 2017; Kragel *et al.*, 2020). However, recent calls for new approaches to address issues that have long nagged the neuroimaging community (multiple comparisons, reverse inference, pattern recognition, basic psychometric development and individual differences) may prove especially beneficial for those in health neuroscience. Indeed, parallel issues regarding statistical inference can be found in other health fields, including psychoneuroimmunology (e.g. El Kissi *et al.*, 2015; Trumpff *et al.*, 2019) and genetics (Flint and Munafò, 2013). Contributions in the current special issue similarly reflect the massive shift toward more sophisticated computational methods in order to test some of the original claims and revisit findings based on small samples from early health neuroscience (Gianaros *et al.*, 2020) and fill in gaps in longstanding models of health (Berkman, 2018; Cosme *et al.*, 2020). To the extent that innovative solutions to ‘big data’ can be refined at the level of the brain, these advances will also be helpful to understanding biomarkers of health risk.

Beyond innovative statistical approaches to the brain and returning to the point to embed the brain in psychological experience, we should remember that humans exist across meaningful experiences and time. The brain’s contribution to health during life transitions remains underexplored (Cardenas *et al*., this issue, highlights how the transition to motherhood might affect the brain). Movement from singlehood to coupledom (and back), for instance, is an active area in health research, but not health neuroscience (Sbarra *et al.*, 2011). Similarly, appreciation for the entire lifespan should continue. Birth is not a blank slate nor are the early years of life (e.g. Gee *et al.*, 2013). As both healthy life expectancy and life expectancy increase worldwide (World Health Organization, 2020), there is also an urgent need to conduct research at later stages of life. Existing longitudinal brain datasets are publicly available for exploration (examples from the current special issue include MIDUS (Alvarez *et al.,* 2020) and AHAB-II (Gianaros *et al.*, 2020; Verstynen *et al.*, 2020)), but an additional way that social, cognitive and affective neuroscientists can contribute to the lifespan perspective is by extending their paradigms to new age groups (especially midlife and later life).

### Think beyond the brain

No one level of analysis—least of all the brain—can tell us all we need to know about health. Only when embedded within the individual’s larger context, behavior, feelings and thoughts and when linked in biologically meaningful and plausible ways to the rest of the body (e.g. predicting a clinical outcome, brain regions that share anatomical connections, etc.) does the brain become useful for understanding health. Others have questioned the value of the brain in health (e.g. Wax, 2017). But the risk of lost opportunities—to understand pathways to and from health, to use brain data to update public opinion and population-level behavior, or make structural changes to healthcare or policy—are too great to abandon the brain so early in its exploration in relation to health.

### Think bigger about health

A major limitation of the current conceptualization of health is that it suggests that health is unidimensional or that it exists on a continuum—from well-being to clinical illness. In reality, health is multidimensional. Physical health, the traditional focus within health neuroscience, exists in the presence of good mental and social health. Taking a multidimensional view of health could help integrate findings across areas showing that similar mechanisms bidirectionally influence each of these health outcomes. As concrete examples, ventromedial prefrontal cortex (VMPFC) activity in response to socio-affective tasks is implicated in mental (e.g. Keedwell *et al.*, 2005), physical (Gianaros and Wager, 2015) and social health (Eisenberger *et al.*, 2011). Similarly, inflammation continues to have tremendous promise as a common mechanism by which psychological experience affects mental, physical (e.g. Dantzer *et al.*, 2008; Lutgendorf and Sood, 2011; Delgado *et al.*, 2018) and social health (Eisenberger *et al.*, 2017; Nusslock and Miller, 2016). Maintaining health neuroscience’s original focus on physical health while accounting for other influential dimensions of health will be important for reaching a consensus about shared mechanisms, points of intervention, and for ultimately gaining a holistic understanding of health.

## Conclusion

Health neuroscience 2.0 is ready for greater innovation at the levels of theory development, psychological experience and its definition of health. With a new lens on the field, we can ask ourselves the big questions that first gave rise to the field: What can the brain tell us about health that will affect how we think and behave individually, socially and as a society? And what preventative or restorative recommendations, interventions and treatment might arise from embedding the brain into current and new models of health? Excluding any part of an individual only takes us further from the goal to understand our complete state of physical, mental and social well-being—the brain is no exception.
